# Low Reflection and Low Surface Recombination Rate Nano-Needle Texture Formed by Two-Step Etching for Solar Cells

**DOI:** 10.3390/nano9101392

**Published:** 2019-09-29

**Authors:** Chia-Hsun Hsu, Shih-Mao Liu, Shui-Yang Lien, Xiao-Ying Zhang, Yun-Shao Cho, Yan-Hua Huang, Sam Zhang, Song-Yan Chen, Wen-Zhang Zhu

**Affiliations:** 1 School of Opto-Electronic and Communication Engineering, Xiamen University of Technology, Xiamen 361024, Chinaxyzhang@xmut.edu.cn (X.-Y.Z.); wzzhu@xmut.edu.cn (W.-Z.Z.); 2 Mechanical and Automation Engineering, Da-Yeh University, Changhua 51591, Taiwan; v0011009@gmail.com; 3 Department of Materials Science and Engineering, Da-Yeh University, Changhua 51591, Taiwan; yunshaojhuo@gmail.com; 4 Industry-University Center, Da-Yeh University, Changhua 51591, Taiwan; 5 Chengyi University College, Jimei University, Xiamen 361021, China; 6 Faculty of Materials and Energy, Southwest University, Chongqing 400715, China; 7 Department of Physics, OSED, Xiamen University, Xiamen 361005, China; sychen@xmu.edu.cn

**Keywords:** black silicon, passivation, metal-assisted chemical etching

## Abstract

In this study, needle-like and pyramidal hybrid black silicon structures were prepared by performing metal-assisted chemical etching (MACE) on alkaline-etched silicon wafers. Effects of the MACE time on properties of the black silicon wafers were investigated. The experimental results showed that a minimal reflectance of 4.6% can be achieved at the MACE time of 9 min. The height of the nanostructures is below 500 nm, unlike the height of micrometers needed to reach the same level of reflectance for the black silicon on planar wafers. A stacked layer of silicon nitride (SiN_x_) grown by inductively-coupled plasma chemical vapor deposition (ICPCVD) and aluminum oxide (Al_2_O_3_) by spatial atomic layer deposition was deposited on the black silicon wafers for passivation and antireflection. The 3 min MACE etched black silicon wafer with a nanostructure height of less than 300 nm passivated by the SiN_x_/Al_2_O_3_ layer showed a low surface recombination rate of 43.6 cm/s. Further optimizing the thickness of ICPCVD-SiN_x_ layer led to a reflectance of 1.4%. The hybrid black silicon with a small nanostructure size, low reflectance, and low surface recombination rate demonstrates great potential for applications in optoelectronic devices.

## 1. Introduction

Silicon reflects a significant amount of incident light on its surface because of its high refractive index of about 3 to 4 in the visible wavelength region. The most common way to reduce the reflection is to texture the silicon surface by iso-etching, which produces micron-scale pyramids on the surface. A dielectric layer is deposited to further reduce the reflection based on the quarter-wavelength design and is therefore optimized only for a given wavelength [1]. Another way is to make nanostructures on a silicon surface (known as black Si [2,3]). A broadband antireflection at a wider acceptance angle than single or multilayer antireflective coatings can be achieved [4,5,6,7,8]. Black silicon can be prepared using reactive ion etching [9], laser–chemical [4,10], electrochemical [5], or metal-assisted chemical etching (MACE) [11,12,13,14]. Some of the techniques produce regular nanostructures, but random textures can reduce reflectance equally well [15]. The MACE process leads to random textures. The metal source of the MACE process can be gold, silver nanoparticles mixed with hydrogen fluorine (HF) and hydrogen peroxide (H_2_O_2_) [6,7]. Due to the sharp nanostructures, the incident light experiences a varying refractive index as a composition of the silicon and the surrounding materials [5,16]. The fraction of the silicon increases when the light goes deeper inside the nanostructures.

Although black silicon has the advantage of low surface reflection, its large surface area leads to a large recombination rate. Long nanostructures with a small diameter lead to very low reflectance, but this does not improve the solar cell performance. Traditional MACE etching for solar cell applications is usually performed on planar wafers, which have high reflectance of about 30–40% in wavelengths of 400–1000 nm. In order to obtain sufficiently low reflectance, the etching depth needs to be deep, and thus, sharp, long nanostructures are produced. These structures increase the difficulties of the doping process and passivation of silicon surface. The emitter region becomes deeper with increasing the nanowire length, and photogenerated carriers at the tip of these nanostructures recombine before being collected at the junction [17]. Although black silicon demonstrates excellent optical properties, simultaneously achieving low reflectance and low surface recombination is getting more desirable and challenging [18].

In this study, a two-step etching process was performed, and it was able to give a low reflectance when the height of the nanostructures is less than 500 nm. The first step was the alkaline etching resulting in a pyramidal surface, and the second step was the MACE that creates needles on the pyramids of the alkaline-etched wafers. Compared to long, sharp nanowires produced by traditional MACE on planar wafers, the small sized nanostructures in turn mean a smaller amount of increase in surface area, and passivation will be relatively easier. In addition, the nanostructures can also be fabricated at a large scale. The MACE time was varied to produce different sizes of the nanostructures. The black silicon was passivated by aluminum oxide and silicon nitride by spatial atomic layer deposition (ALD) and plasma enhanced chemical vapor deposition (PECVD), respectively. The reflectance and the passivation quality of the black silicon wafers are presented and discussed. Low reflectance and high passivation on silicon wafers with small-sized nanostructures can be achieved.

## 2. Materials and Methods

**Figure 1:**
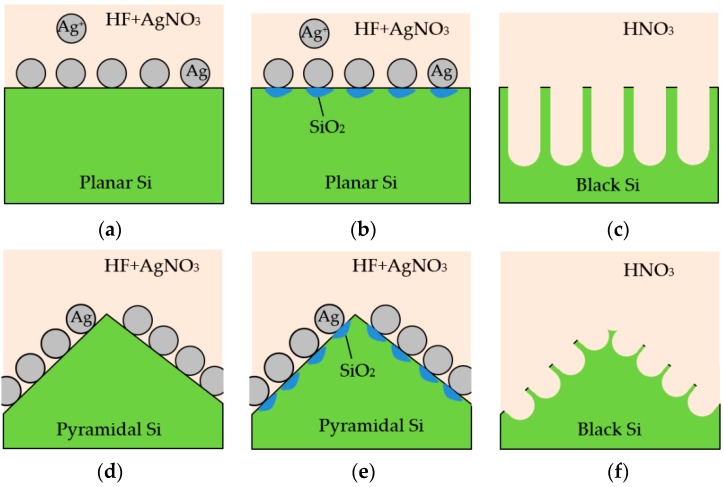
Schematic diagram of the metal-assisted chemical etching (MACE) process: (**a**) silver particles on the planar wafer; (**b**) local oxidation of Si on the surface; (**c**) sharp and deep needles formed after removing Ag particles by HNO_3_; (**d**) silver particles on the pyramidal surface of an alkaline-etched silicon wafer; (**e**) silicon oxidation on the pyramidal surface; and (**f**) small-size needles formed on the pyramidal surface.

Boron-doped crystalline silicon wafers with a thickness of 200 μm and a resistivity of 1 Ω-cm were used as substrates. The conventional MACE process was usually performed on planar wafers as shown in Figure 1a–c. When the Ag^+^ ions were in contact with the silicon surface, they reduced to Ag particles and ejected holes into silicon, catalyzing local silicon oxidation beneath the Ag particles. The silicon oxidation was then dissolved by HF. The MACE process can be expressed by:

Ag^+^ → Ag + h^+^, (1)

Si + 2H_2_O + 4h^+^ → SiO_2_ + 4H^+^, (2)

SiO_2_ + HF → H_2_SiF_6_ + H_2_O (3)

where h^+^ is the electronic hole. The MACE process was finished by removing the Ag particles from the silicon surface by HNO_3_. Very sharp and deep structures were needed to reach sufficiently low reflectance. In this study, the texturization of the silicon wafers consisted of two steps. The wafers were firstly etched in alkaline solution consisting of 20 g of potassium hydroxide (KOH), 50 mL of isopropyl alcohol (IPA), and 1 L of deionized water at a temperature of 80 °C for 40 min to form randomly distributed pyramids in a micrometer scale on the silicon wafer surface. The size of the pyramids is not uniform, but generally, the base width and height of the pyramids are a few microns with an angle of about 54°. The wafer thickness was reduced to 170 μm. The wafers were then etched by a mixture of 0.6 M HF and 0.03 M AgNO_3_ solutions at 40 °C for 1 to 9 min to produce nanostructures on the pyramids. Figure 1d–f shows the schematic of the two-step texturization process. Shorter structures were able to significantly reduce the reflectance as compared to the conventional MACE process. After the MACE etching process, the wafers were cleaned by a standard Radio Corporation of America process, followed by a dip in HF for 1 min. For the passivation of the silicon wafers, an aluminum oxide (Al_2_O_3_) layer was deposited using a spatial ALD system (model AL_2_O_3_, Henghau Enterprise Co., Ltd., Taiwan) with trimethylaluminum (TMA) and water as the precursors. The gap between the ALD injector and movable substrate was 60 μm. The temperatures of the TMA and H_2_O bubblers were 17.5 °C and 27 °C, respectively. The pipe temperature was 40 °C, which was higher than the bubblers temperature to avoid condensation. The substrate temperature was 150 °C. An inert gas N_2_ was used as a curtain to isolate TMA and H_2_O over the substrate. The growth per cycle of the Al_2_O_3_ was 0.17 nm/s, within the typical range reported in literature [19]. Afterwards, a silicon nitride (SiN_x_) layer was deposited using radiofrequency inductively coupled plasma chemical vapor deposition (ICPCVD SI-500 D, SENTECH, Berlin, Germany) with a gas mixture of trimethylsilane and ammonia. The substrate temperature was 120 °C. The radiofrequency power was 1200 W. The deposition pressure was 5 mTorr. The ALD and ICPCVD processing parameters can also be found elsewhere [20]. For the characterization, the morphologies of the films were observed using a scanning electron microscope (SEM, JSM-7800F, JEOL, Tokyo, Japan). The reflectance of the wafers was measured using a UV-visible spectrometer (U-3900, Hitachi, Marunouchi, Japan). The injection-level dependent minority carrier lifetime of the wafers was measured using a lifetime tester (WCT-120, Sinton Instruments, Boulder, CO, USA). The cross-sectional images of the samples were obtained with a transmission electron microscope (TEM, JEM2100, JEOL, Tokyo, Japan) at 200 kV.

## 3. Results and Discussion

**Figure 2:**
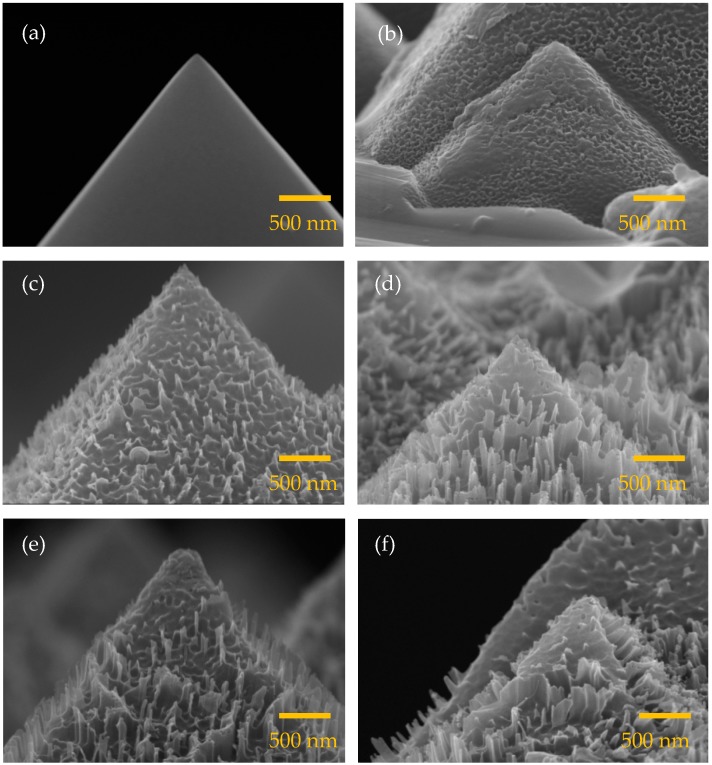
Scanning electron microscope (SEM) images for the alkaline-etched silicon wafers with the metal-assisted chemical etching (MACE) process for (**a**) 0, (**b**) 1, (**c**) 3, (**d**) 5, (**e**) 7, and (**f**) 9 min.

Two etching processes were applied to wafers for the surface texturing, which were alkaline etching to form randomly-distributed pyramids at a micrometer scale and MACE to further form needle-like structures in nanoscale. The etching time of the MACE process was varied. Figure 2 shows the SEM images of the silicon wafers without and with different MACE times. The wafer without MACE in turn means that only alkaline etching was performed, and thus, a typical micrometer-scale pyramidal structure is formed on the wafer surface as shown in Figure 2a. There is no any substructure observable on the pyramid surfaces. The morphologies of the wafers change when the MACE time increases. At a MACE time of 1 min (Figure 2b), the wafer surface shows many shallow cavities, evidencing the removal of the silicon. The MACE time of 3 min produces pronounced needles on the pyramid surfaces as shown in Figure 2c. The size of the needles is not uniform, but the maximum height of the needles is roughly estimated to be 270 nm. Further increasing the MACE time remarkably enhances the development of the nanostructures, but with a height below 500 nm (Figure 2d–f). It is also noted that the some of the needles seem to connect to each other. In literature, the black silicon structures are mostly made by starting with a planar/polished wafer. Needles with a height at a micrometer level are needed in order to have sufficiently low reflectance [21,22]. Crystalline silicon solar cells usually have a shallow p–n junction with a junction depth of a few hundred nanometers from the front surface [23,24,25]. If the size of the nanostructures of the black silicon is larger than the junction depth, then the emitter diffusion process needs to be performed after the MACE process to avoid destroying the p–n junction. However, diffusion on such a silicon wafer with sharp needles is difficult. The nanostructure size in this study is not beyond the junction depth, and therefore, a standard diffusion process can be performed, followed by the MACE process.

**Figure 3:**
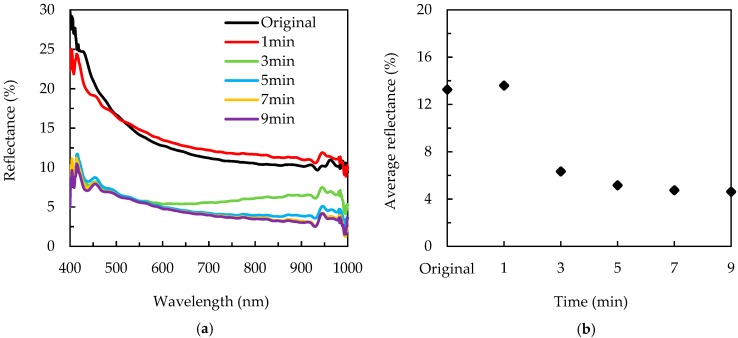
(**a**) Reflectance spectra and (**b**) average reflectance for the alkaline-etched silicon wafers with a different MACE time. The label “Original” refers to the sample with alkaline etching but without the MACE process.

The reflectance spectra over 400–1000 nm of the alkaline-etched silicon wafers with different MACE time are shown in Figure 3a. The reflectance of the original wafer (with alkaline etching but without the MACE process) is in the range between 10–30%, similar to the wafer with a MACE time of 1 min. The wafers with MACE times of 3–9 min have a nearly identical reflectance in the short-wavelength region (400–550 nm), while the reflectance at the mid- to long-wavelengths (600–1000 nm) decreases with the MACE time. This result clearly evidences that the nanostructures of the wafers can reduce the reflectance in the whole investigated wavelengths as compared to the wafer with the pyramidal surface. The average reflectance of the wafers is shown in Figure 3b. The values of the average reflectance of the wafers without MACE and with MACE time of 1 min are around 13%, a typical value for a wafer with a pyramidal surface. The average reflectance drops to 6.3% at a MACE time of 3 min. This reduction results from the presence of the needle-like structures. The wafers without and with a MACE time of 1 min show a silver-grey color, while the wafer with a MACE time of 3 min has a dark grey or nearly black color. The average reflectance further reduces from 6.3% to 4.6% when the MACE time increases from 3 to 9 min. Further increasing the etching time (not shown here) leads to slight or negligible reduction in average reflectance. This saturation behavior can be associated with the reduced concentration of HF in the etching solution. During the MACE process, the HF plays the role of removing silicon oxide. The amount of HF is consumed, and the removal released in the solution further decreases the concentration of HF. The etching process is thus expected to slow down and eventually stop. The MACE process performed on pyramidal surfaces of silicon wafers can achieve both low reflection and a small structure size.

**Figure 4:**
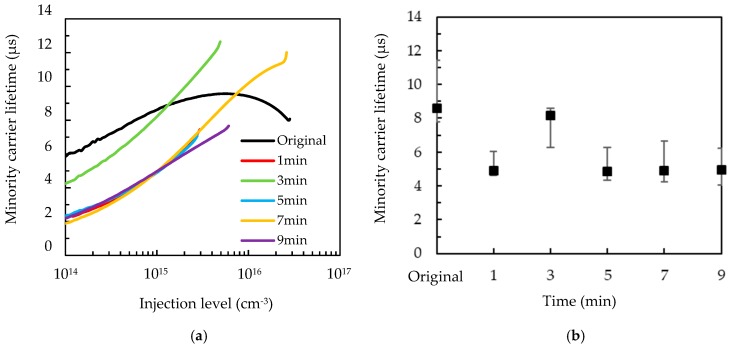
(**a**) Injection-level dependent minority carrier lifetime; and (**b**) average minority carrier lifetime at the injection level of 3 × 10^15^ cm^−3^ for the alkaline-etched silicon wafers with a different MACE time.

Figure 4a shows the injection-level dependent minority carrier lifetime of the wafers with a different MACE time. The lifetime values at the injection level of 3 × 10^15^ cm^−3^ are shown in Figure 4b for comparison. Five samples were prepared for each MACE time, and the average values and error bars are indicated. It can be seen that the minority carrier lifetime of the original wafers (i.e., before MACE etching) already fluctuates from 7.8 to 11.4 μs with an average of 8.6 μs, and this could cause the variation of the lifetime of the wafers after the MACE process. However, the lifetime fluctuation of the original wafers is small, and thus, the impact is obvious for very low carrier lifetime values. For the passivated wafers with high carrier lifetime values, the impact is insignificant. The wafers with MACE times of 1–9 min show the minority carrier lifetime values in a range of 4–8.2 μs. All the wafers with MACE show a slight decrease in minority carrier lifetime. It has been demonstrated that the minority carrier lifetime decreases by increasing the front surface area [26,27]. The MACE processes lead to dangling bonds and dislocations that also result in a severe Shockley–Read–Hall recombination [18]. The small size of the developed nanostructures in the present study is helpful for mitigating the adverse effect.

**Figure 5:**
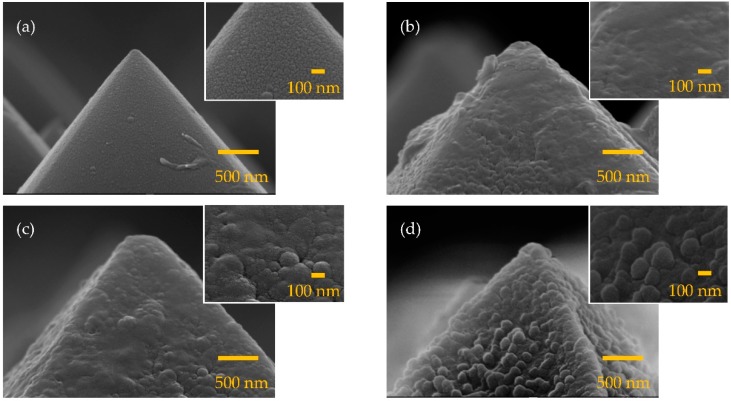

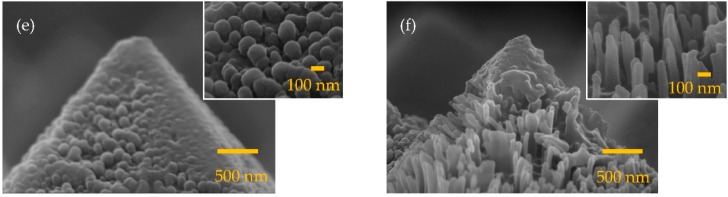
SEM images of the SiN_x_/Al_2_O_3_-passivated wafers with MACE times of (**a**) 0; (**b**) 1; (**c**) 3; (**d**) 5; (**e**) 7; and (**f**) 9 min. The insets are a close-up view of the wafer surfaces.

It is required to further lower the reflectance and passivate the wafers. A stacked layer of SiN_x_/Al_2_O_3_ is a good choice for use as antireflective layer and passivation layer [28,29]. Figure 5 shows the SEM images of the passivated wafers with a different MACE time. The thicknesses of the SiN_x_ and Al_2_O_3_ are respectively selected to be 90 and 15 nm. The insets of the figures are the close-up views. For the wafers without the MACE process, it can be seen that the passivation layer is smoothly covered on the wafer surface. A relatively rigid surface morphology can be observed for a MACE time of 1 min. The wafer with a MACE time of 3 min shows some rounded structures on the wafer surface, which is different to the needle-like structures before the deposition of the passivation layer. This change in the morphology indicates a low conformity of the passivation layer deposition. The rounded structures are more pronounced at MACE times of 5 and 7 min. At a MACE of 9 min, the wafer has needle-like structures, similar to the morphology before the passivation layer deposition. It is assumed that the passivation layer may either be conformally or hardly deposited. As the height of the nanostructures developed by the MACE process at 9 min is the largest, the passivation layer is more reasonable to be hardly deposited on the wafer rather than a conformal deposition.

**Figure 6:**
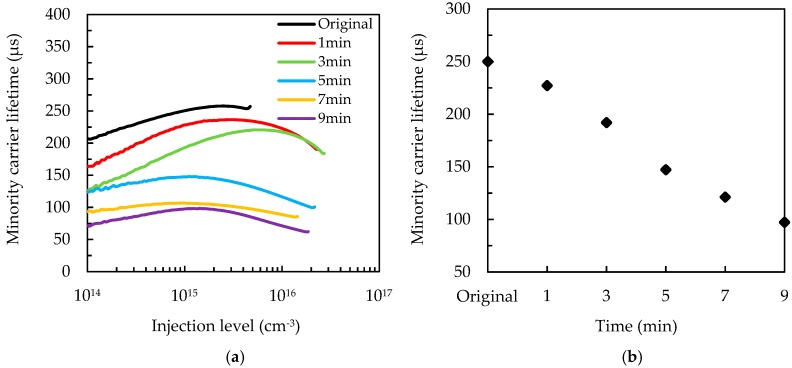
(**a**) Injection-level dependent minority carrier lifetime, and (**b**) average minority carrier lifetime at the injection level of 3 × 10^15^ cm^−3^ for the SiN_x_/Al_2_O_3_-passivated silicon wafers with a different MACE time. The labeled “Original” refers to the passivated wafer with only alkaline etching but without the MACE process.

To investigate the passivation quality, the injection-level dependent minority carrier lifetime values of the wafers after passivation are shown in Figure 6a, and the lifetime values at the injection level of 3 × 10^15^ cm^−3^ are shown in Figure 6b. The chemical passivation and field-effect passivation provided by the SiN_x_/Al_2_O_3_ stacked layer reduce the surface recombination rate and increase the minority carrier lifetime as compared to the samples without the passivation layer. The original wafer (without the MACE process) has the highest lifetime value of around 250 μs, corresponding to a surface recombination rate of 34 cm/s. The original wafer has pyramids without sharp needles, and therefore, the passivation layer covers the surface well. As the MACE time increases from 1 to 9 min, the wafer lifetime decreases from 227.3 to 97.6 μs, and the surface recombination rate increases from 37.4 to 87.1 cm/s. This indicates that the passivation layer is not fully deposited on the wafers, especially for those connected needle structures. The SiN_x_/Al_2_O_3_ films can hardly cover the boundaries or sidewalls of two narrow-spaced needles, leaving the dangling bonds unpassivated. Nevertheless, the currently standard c-Si solar cells use SiN_x_ single layer as the passivation layer of the front surface of the wafers, and the surface recombination rate is mostly in the level of 10^2^ cm/s [30,31]. The low surface recombination rates for the samples in this study demonstrate a high surface passivation quality because of the ALD Al_2_O_3_ layer.

**Figure 7:**
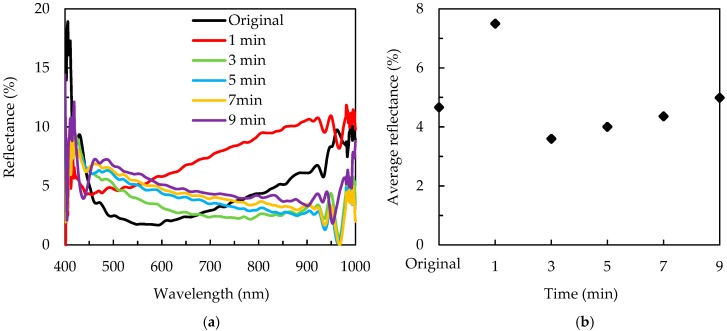
(**a**) Reflectance spectra, and (**b**) average reflectance for the SiN_x_/Al_2_O_3_ passivated silicon wafers with a different MACE time.

The reflectance spectra of the passivated wafers with different MACE times are shown in Figure 7a. The wafers without the MACE process and with a MACE time of 1 min show the curves with a single minimum reflectance. From the SEM image (Figure 2b), the 1 min MACE etching does not produce needle structures, while it creates shallow cavities and smoothens the tips and edges of the pyramids. This is a possible reason for the higher reflectance of the 1 min MACE etched sample with the SiN_x_/Al_2_O_3_ stack compared to the others. This is a typical shape of a reflectance spectrum curve for an antireflective layer deposited on silicon. The wafers with MACE times of 3–9 min exhibit a low reflectance in broad wavelengths. The average reflectance values are shown in Figure 7b. For the alkaline-etched wafer with SiN_x_/Al_2_O_3_, the reflectance is 4.7%. A minimal reflectance of 3.6% is reached at a MACE time of 3 min. The reflectance increases with further increase of the MACE time. The increase in reflectance for long MACE times is presumably due to the poor coverage of the SiN_x_/Al_2_O_3_ layers. The wafer with a MACE time of 9 min has a reflectance of 4.9%, nearly same as before the deposition of SiN_x_/Al_2_O_3_ layers. This also confirms that the antireflection layer is hardly coated on the wafer. It is known that ALD is able to provide a very high conformity deposition, and thus, the coating problem should arise from the PECVD SiN_x_ deposition. Other research groups also reported that the PECVD has difficulties of gap filling or depositing void-free films on high aspect ratio structures [32,33].

**Figure 8:**
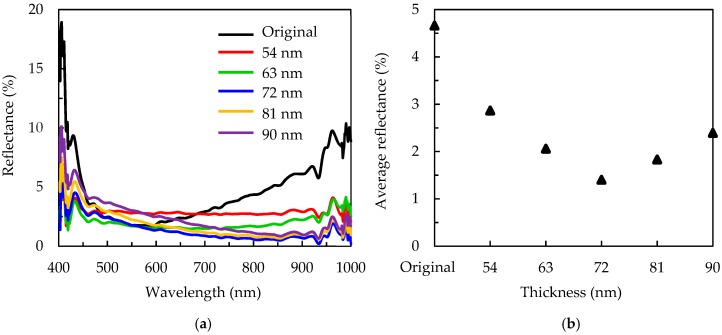
(**a**) Reflectance spectra, and (**b**) average reflectance for the 3 min MACE-treated silicon wafers covered with different SiN_x_ thickness. The labeled “Original” refers to the pyramidal silicon wafer (without the MACE process) with the 90 nm SiN_x_/15 nm Al_2_O_3_ stacked layer.

The thickness of the SiN_x_ is varied from 54 to 90 nm to further optimize the reflectance for the 3 min MACE etched sample. The thickness of the Al_2_O_3_ is kept to 15 nm. Figure 8a shows the reflectance spectra for the wafers with different SiN_x_ thickness. The labeled “Original” refers to the pyramidal wafer with the 90 nm SiN_x_/15 nm Al_2_O_3_ stacked layer. The average reflectance is shown in Figure 8b. The refractive indices of the SiN_x_ and Al_2_O_3_ are 1.8 and 1.7, respectively. For conventional silicon crystalline solar cells, the SiN_x_ single antireflective layer should have an optimal thickness of 80–90 nm according to the quarter-wavelength formula to achieve a reflectance minimum at 550–600 nm. The average reflectance over 400–1000 nm of the 90 nm SiN_x_-covered pyramidal silicon wafers is around 4% (not shown). In order to have high passivation, the pyramidal wafer is covered with not only 90 nm SiN_x_ but 15 nm Al_2_O_3_, and this in turn means that the total thickness of the antireflective layer becomes larger, resulting in a slight increase of reflectance to 4.6%. By adjusting SiN_x_ thickness, the reflectance of the 3 min MACE etched wafer decreases to its minimum of 1.4% at the SiN_x_ thickness of 72 nm. The total thickness of SiN_x_/Al_2_O_3_ is 87 nm.

**Figure 9:**
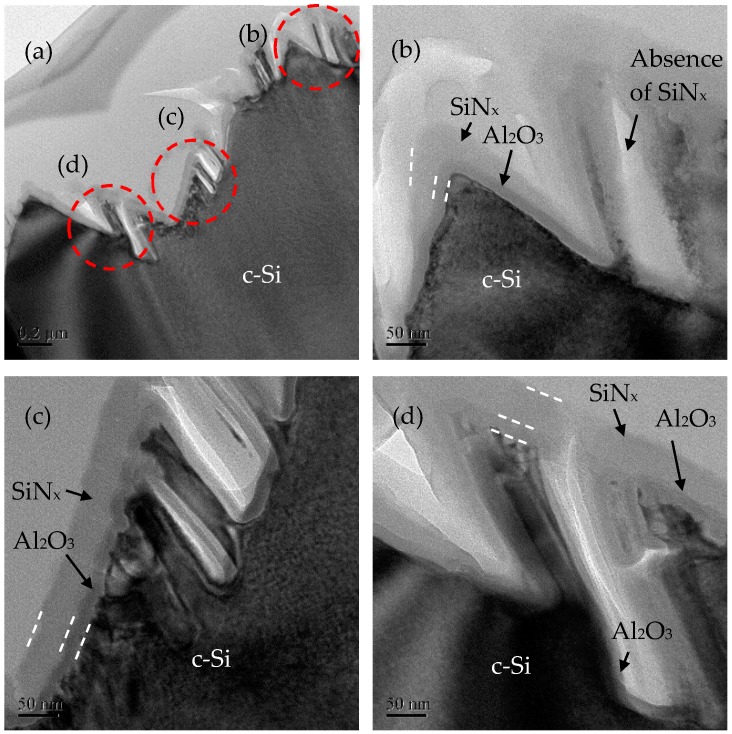
(**a**) Cross-sectional transmission electron microscope (TEM) images for black silicon with a MACE time of 3 min and SiN_x_/Al_2_O_3_ passivation layers. The red circles are different locations in the black silicon wafer, and (**b**–**d**) are their close-up views.

Figure 9 shows the cross-sectional images of the 72 nm SiN_x_/15 nm Al_2_O_3_-passivated pyramidal silicon wafers with a MACE time of 3 min. The nanostructures with heights of 200–300 nm can be seen in Figure 9a. Close-up views are taken for some regions labeled as b–d. In region b (Figure 9b), the SiN_x_/Al_2_O_3_ is covered on the top of the pyramid. The SiN_x_ is the thickest on the top, and the thickness decreases obviously at the valley. The Al_2_O_3_ shows a uniform thickness when covering on the wafer. It is noted that for the nanostructures on the right-hand side, the Al_2_O_3_ can still be well deposited, whereas the SiN_x_ only covers on the top of the nanostructure. Thus, there is an absence of SiN_x_ at two nanostructures, as appearing in white-grey color in the image. Similar results can be observed in Figure 9c,d. At the sidewall of the pyramidal surface, both of the Al_2_O_3_ and SiN_x_ can be well deposited, but the SiN_x_ cannot fill the gap between the nanostructures. These empty spaces indicate that ALD passivation is necessary for black silicon; otherwise, the traditional PECVD films are hardly covered on the surfaces.

The black silicon with a MACE time of 3 min and 72 nm SiN_x_/15 nm Al_2_O_3_ passivation demonstrates a reflectance of 1.4% and a surface recombination rate of 43.6 cm/s. Currently, conventional crystalline silicon mainstream PV products have a pyramidal front surface with a SiN_x_ single layer as an antireflective layer and passivation layer. The average reflectance is around 4%, and the surface recombination rate is at a level of 10^2^ cm/s. These values could be used for estimating the gain in short-circuit current density (J_sc_) and open-circuit voltage (V_oc_) in the device level, as given by [34,35]:

(4) $$J_{\mathrm{sc}}=\int \left[1-R\left(\lambda \right)\right]IQE\left(\lambda \right)S\left(\lambda \right)d\lambda$$

(5) $$V_{\mathrm{oc}}=\frac{kT}{q}\ln\left(\frac{J_{sc}}{J_{0b}+J_{0e}}+1\right)$$

where *λ* is the wavelength, *R* is the reflectance, *IQE* is the internal quantum efficiency, *S* is the solar spectrum, *kT*/*q* is the thermal voltage, *J*_0*b*_ is the base dark current, and *J*_0*e*_ is the emitter dark current. For simplification by assuming that the MACE process only influences the surface of the wafers and does not affect *IQE*, the variation of J_sc_ can be evaluated by *R*. Equation (4) gives that the *J_sc_* for the black silicon can increase by 2.6% compared to that for the traditional c-Si. For the variation of V_oc_, it is determined by *J*_0*e*_ as the black silicon nanostructures are designed on the light-receiving side (emitter side). *J*_0*e*_ is given by [36]:

(6) $$J_{0e}=\frac{qn_{i}{}^{2}W}{2}\frac{1}{N_{B}+∆n}\frac{1}{\tau_{suf}}$$

where *q* is the electron charge, *n_i_* is the intrinsic carrier concentration, *W* is the wafer thickness, *N_B_* is the bulk doping concentration, ∆*n* is the excess carrier concentration (3 × 10^15^ cm^−3^), and *τ_suf_* is the lifetime associated with the surface recombination. By assuming the infinite bulk lifetime, *τ_suf_* is equal to the measured effective minority carrier lifetime. The calculated *J*_0*e*_ is about 53 fA/cm^2^ for black silicon and 113 fA/cm^2^ for the conventional c-Si solar cell. It is considered that only the surface property is affected, while the others are not influenced by the MACE process. Thus, from Equations (5) and (6), the V_oc_ of the black silicon increases by 2% compared to that of the conventional cells. Another way to evaluate the open-circuit voltage is the one-sun implied V_oc_ obtained from the WCT-120 lifetime measurement. Table 1 lists the implied V_oc_ for different nanostructures passivated by the SiN_x_/Al_2_O_3_ stack. The labeled “Reference” corresponds to the SiN_x_ single layer-passivated pyramidal wafer. The implied V_oc_ of the 3 min MACE etched wafer is about 2.1% higher than that of the reference sample, and the gain is in agreement with that evaluated by using *J*_0*e*_. Overall, as the solar cell conversion efficiency is the multiplication of V_oc_, J_sc_ and fill factor, the black silicon proposed in this work is helpful for increasing the c-Si solar cell efficiency.

## 4. Conclusions

Needle-like nanostructures were prepared on pyramidal surfaces of c-Si using MACE processes. The reflectance can greatly reduce to below 5% when the height of the needle-structures is below 500 nm, typically smaller than the junction depth of c-Si solar cells. A SiN_x_/Al_2_O_3_ stacked layer is covered on the black silicon surface as an antireflective and passivation layer. The ALD Al_2_O_3_ film is found to be conformally deposited on the black silicon, while the PECVD SiN_x_ film shows a poorer coverage or is even hardly coated between two needle-like structures. The optimum MACE time is 3 min, and the passivated wafer exhibits a nanostructure height of less than 300 nm, the lowest reflectance of 1.4% and a low surface recombination rate of 43.6 cm/s. Compared to the currently standard c-Si solar cells with a reflectance of about 4% and a front surface recombination rate at 10^2^ cm/s level, the black silicon technique proposed in this work is helpful for improving the solar cell conversion efficiency.

## Figures and Tables

**Table 1:** One-sun implied V_oc_ of the SiN_x_/Al_2_O_3_-passivated silicon wafers with different MACE time. The labeled “Reference” corresponds to the SiN_x_ single layer-passivated pyramidal wafer.

MACE Time (min)	Implied V_oc_ (mV)
Reference	676
1	692
3	690
5	688
7	684
9	679
